# Myocarditis Following Immune Checkpoint Inhibition With Pembrolizumab: Management in a Context of Steroid Intolerance

**DOI:** 10.1016/j.cjco.2022.07.002

**Published:** 2022-07-11

**Authors:** Laura L. Onderko, Ross Heinrich, Katalin Gosling, Tim Downs, Maxwell Eyram Afari

**Affiliations:** a Department of Cardiology, Maine Medical Center, Portland, Maine, USA; b Department of Internal Medicine, Maine Medical Center, Portland, Maine, USA

## Abstract

Immune checkpoint inhibitors (ICIs) are a major advance in oncology and have become first- or second-line therapy for over 50% of oncology patients. ICI-associated myocarditis is a complication that, although rare, has a high mortality rate. We present a case of ICI-associated myocarditis presenting as complete heart block. Traditional treatment with high-dose steroids was abandoned in this case, owing to steroid-induced psychosis. Alternative treatment with immunomodulators was initiated with a good response. This case highlights the variable presentation of ICI-associated myocarditis. As use of ICIs continues to expand, an understanding of their adverse reactions and best treatments will be needed.

## Résumé

Les inhibiteurs des points de contrôle immunitaire (IPCI) constituent un progrès majeur dans le domaine de l’oncologie, et sont maintenant utilisés comme traitement de première ou de deuxième intention pour plus de 50 % des patients en oncologie. La myocardite associée aux IPCI est une complication rare, mais dont le taux de mortalité est élevé. Nous présentons ici un cas de myocardite associée aux IPCI caractérisé par un bloc cardiaque complet. Le traitement classique par doses élevées de stéroïdes a dû être abandonné en raison d’une psychose provoquée par les stéroïdes. Un autre traitement par des immunomodulateurs a été amorcé et a produit une réponse satisfaisante. Ce cas permet de souligner la variété des tableaux cliniques des myocardites associées aux IPCI. Alors que l’utilisation des IPCI continue d’augmenter, une meilleure compréhension des réactions indésirables et des options pour leur prise en charge seront nécessaires.

Immune checkpoint inhibitors (ICIs) represent a major advance in the field of immunotherapy and have changed the standard of care for many cancer types. James P. Allison and Tasuku Honjo were awarded the Nobel Prize in Physiology or Medicine in 2018 for their work in immune checkpoint blockade. Over the past decade, ICIs have become first- or second-line therapy for over 50% of patients diagnosed with cancer.^1^

Immune checkpoints are regulatory pathways that attenuate the immune response and are upregulated by tumour cells in order to evade attack.^2^ ICIs target these immune checkpoints to promote an anti-tumour immune response.^2^ Histologic staining of tumours that is positive for programmed death ligand-1 is predictive of efficacy of these agents. However, the activation of this immune response is not specific to tumour cells and therefore can lead to autoimmune-like adverse effects affecting various organ systems.^2^ Although ICI-associated cardiotoxicities are not the most common type of immune-related adverse events (IRAEs), ICI-induced cardiotoxicities can be fatal.^3^ As ICI therapy is becoming more common, awareness of ICI-associated cardiovascular adverse events needs to increase in order to avoid delay in diagnosis, treatment, and management.

## Case

A 67-year-old woman with a history of hypertension (on metoprolol tartrate 50 mg twice a day and lisinopril 5 mg daily) and depression (on paroxetine 30 mg daily) was diagnosed with stage IV recurrent endometrial cancer. Following 2 cycles of pembrolizumab, with doses spaced 2 weeks apart, she presented with 1 week of fatigue, shortness of breath on exertion, and a presyncopal episode. She was found to be in complete heart block. A transvenous pacemaker was placed, and she was admitted to the cardiac intensive care unit for further management and workup.

Her oncologic history included a prior history of endometrial cancer, for which she underwent total abdominal hysterectomy and bilateral salpingo-oophorectomy in 2011, followed by radiation therapy in 2015 for recurrence of disease with metastasis. She was subsequently treated with the aromatase inhibitor anastrozole, to reduce endogenous estrogen exposure, and the monoclonal antibody denosumab to reduce cytokine-mediated bone turnover in the presence of reduced estrogen. A restaging positron emission tomography scan in 2016 showed a new area of metastasis requiring further radiation therapy. In 2021, she was started on ICI therapy with pembrolizumab, and at the time of presentation, she had completed cycle 2 and was 34 days from initiation of ICI treatment.

**Figure 1:**
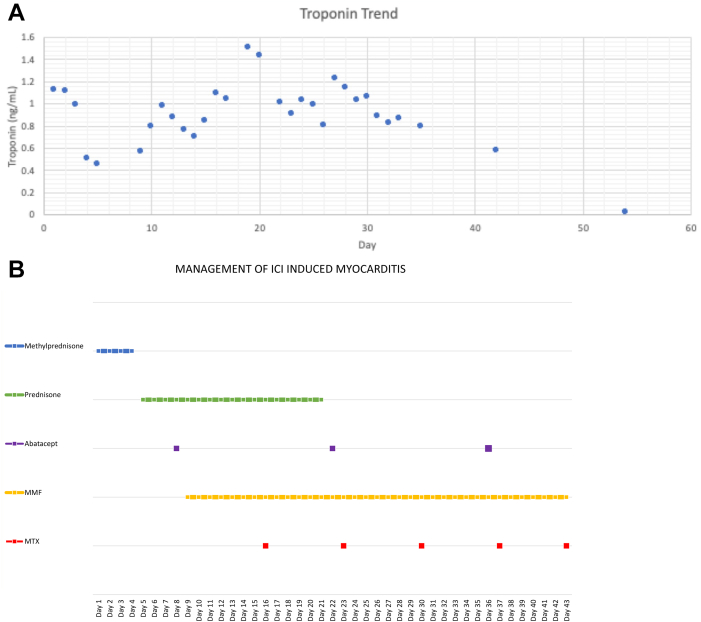
Troponin concentration trend and timeline of medications. (**A**) Shown is the troponin T concentration trend, with peak troponin of 1.4 ng/mL (normal: < 0.03 ng/mL). (**B**) Timeline of medications given in management of immune checkpoint inhibitor (ICI)-induced myocarditis. Initially treated with methylprednisolone, then prednisone, which was replaced with methotrexate, abatacept, and mycophenolate mofetil (MMF) after the patient developed steroid-induced psychosis necessitating treatment adjustment.

Further workup as an inpatient showed an elevated troponin T concentration (a high sensitivity troponin assay was not available) (Fig. 1A), which peaked at 1.4 ng/mL (1400 ng/L; normal reference: < 0.03 ng/mL). A potential etiology for her conduction disease was ischemia. She underwent a coronary angiogram, which showed no epicardial coronary artery disease. Her echocardiogram showed preserved biventricular function and no structural heart disease. She did not have evidence of underlying conduction disease, on an electrocardiogram done just prior to starting ICI therapy, to suggest senile degeneration of the conduction system or progression of an infiltrative process or radiation-induced conduction disease. Given the recent initiation of ICI therapy, suspicion was high that her presentation was most consistent with ICI-associated myocarditis. No cardiac magnetic resonance imaging was performed, due to the continued need for cardiac pacing with a transvenous pacemaker and subsequently a permeant pacemaker. Endomyocardial biopsies were obtained and were consistent with myocarditis, showing edematous myocardium with significant myocyte damage, including cytoplasmic vacuolization and myocyte necrosis. Immunohistochemical analysis showed clusters of cytotoxic cluster of differentiation (CD)8+ T-lymphocytes and staining for programmed death ligand-1 in areas of cellular damage (Supplemental Fig. S1).

The patient was initially treated with intravenous methylprednisolone 1 gram daily for 4 days and then transitioned to 1 mg/kg prednisone. However, due to the development of severe steroid-related psychosis, she was quickly weaned from steroids within 2 weeks. Her adverse reaction to steroids precluded their further use, and therefore, alternative immunomodulators were employed. Based on review of the literature, and specifically the case report from Salem et al. and the treatment paradigm outlined by Bermas et al., she was transitioned to the CD80/86 binding T cell inhibitor—abatacept 500 mg every 2 weeks.^4^^,^^5^ Troponin concentrations were monitored daily while she was an inpatient, and the dose of abatacept was increased to 1000 mg after the first 2 doses, owing to increasing troponin concentrations. She ultimately received 3 doses of abatacept. Following the first dose of abatacept, she was also started on mycophenolate mofetil (MMF), 750 mg orally twice a day (dose reduced from 1000 mg due to leukopenia), and methotrexate 15 mg weekly for 6 weeks as adjunctive treatment, as shown in Figure 1B. These medications were both started due to intolerance of prednisone and persistence of complete heart block, in addition to an increase in cardiac arrhythmias on telemetry, specifically non sustained ventricular tachycardia.

Prior to discharge, a dual chamber pacemaker was placed due to nonresolution of complete heart block. She remained an inpatient for a total of 6 weeks in the cardiology department. Following hospital discharge, she completed the prescribed 6 weeks total of methotrexate and MMF. A repeat check of troponin concentration was done when she was an outpatient, to monitor response to treatment, and was within normal range. She was transitioned to fulvestrant, an estrogen receptor downregulator, for treatment of her malignancy. She was not restarted on ICI therapy. She continued to follow up with cardiology, and interrogation of her device at 9 months after her initial presentation revealed continued complete pacemaker dependence.

## Discussion

Although ICIs have proven to be effective against many cancers, their use is associated with IRAEs, and the T lymphocyte activation seen with ICIs is not specific for cancer cells. This activation can lead to autoimmune-like adverse events that involve various organ systems, and IRAEs have been reported to occur in 60%-80% of patients treated with ICIs.^3^ Cardiovascular adverse events that have been reported include myocarditis, cardiomyopathy, arrhythmias, pericarditis, vasculitis, atherosclerotic cardiovascular events, Takotsubo syndrome, and venous thromboembolism.^3^ Myocarditis has the highest mortality rate, estimated to be as high as 50%, and occurs in about 1% of patients treated with ICIs.^3^

The exact mechanism of ICI-related myocarditis is not completely understood, but it is believed to be related to the ICI inhibition of pathways that protect cardiomyocytes against autoreactive lymphocytes and therefore allow for activation of T cells targeting cardiomyocytes.^6^ Histologically, endomyocardial biopsies show immune infiltrates, specifically T lymphocyte infiltration expressing CD8^+^.^7^

Overall, the majority of cases occur early in the treatment course, occurring within the first 1 or 2 treatment cycles.^8^ Currently, no guidelines have been developed for baseline screening for ICI-associated myocarditis in patients being treated with ICIs. Workup includes laboratory studies with cardiac biomarkers, including troponin, creatine kinase-myocardial band, and N-terminal pro-brain natriuretic peptide, as well as echocardiography, although approximately 50% of patients will have preserved left ventricular ejection fraction.^7^ Further investigations can include cardiac magnetic resonance imaging and endomyocardial biopsy.

Treatment includes discontinuation of ICIs and initiation of corticosteroids.^3^ Despite the increasing use of ICIs, no evidence-based guidelines have been established for management of their known IRAEs. Beyond glucocorticoids, which are typically considered the first line of treatment, alternative treatments are based on case reports.^4^ Troponin concentrations are monitored serially to assess response to treatment, with escalation if the patient fails to respond or worsens.^7^

Our case report characterizes the response to combination immunosuppressive therapy of abatacept, methotrexate, and MMF in a patient with steroid-induced psychosis. This information can be used to guide similar cases, and treatments should be reviewed in a larger analysis. Despite the unlikelihood of large trials, any research into the efficacy of immunosuppressive combinations used as therapy for ICI-associated myocarditis would benefit the management of this potentially growing group of patients.

## Conclusion

Alternative treatments, besides high-dose steroids for ICI-associated myocarditis, warrant further investigation, and prospective trials are needed to determine if other treatment options are superior.

**Novel Teaching Points**

• ICI-associated myocarditis can have variable presentation, including arrhythmias, conduction disorders, and heart failure.
• Although steroids are traditionally the first-line treatment for ICI-associated myocarditis, other immunomodulators can be utilized in patients who cannot tolerate steroids, and these treatments may prove to be superior once more research is conducted.
