# Supplementary material for: Myocarditis Following Immune Checkpoint Inhibition With Pembrolizumab: Management in a Context of Steroid Intolerance
Source: CJC Open. 2022 Jul 11;4(10):854–7. doi: 10.1016/j.cjco.2022.07.002 (PMC9568688; doi:10.1016/j.cjco.2022.07.002)
Supplement: Supplemental Fig. S1 [file mmc1.pdf]

SUPPLEMENTARY MATERIAL

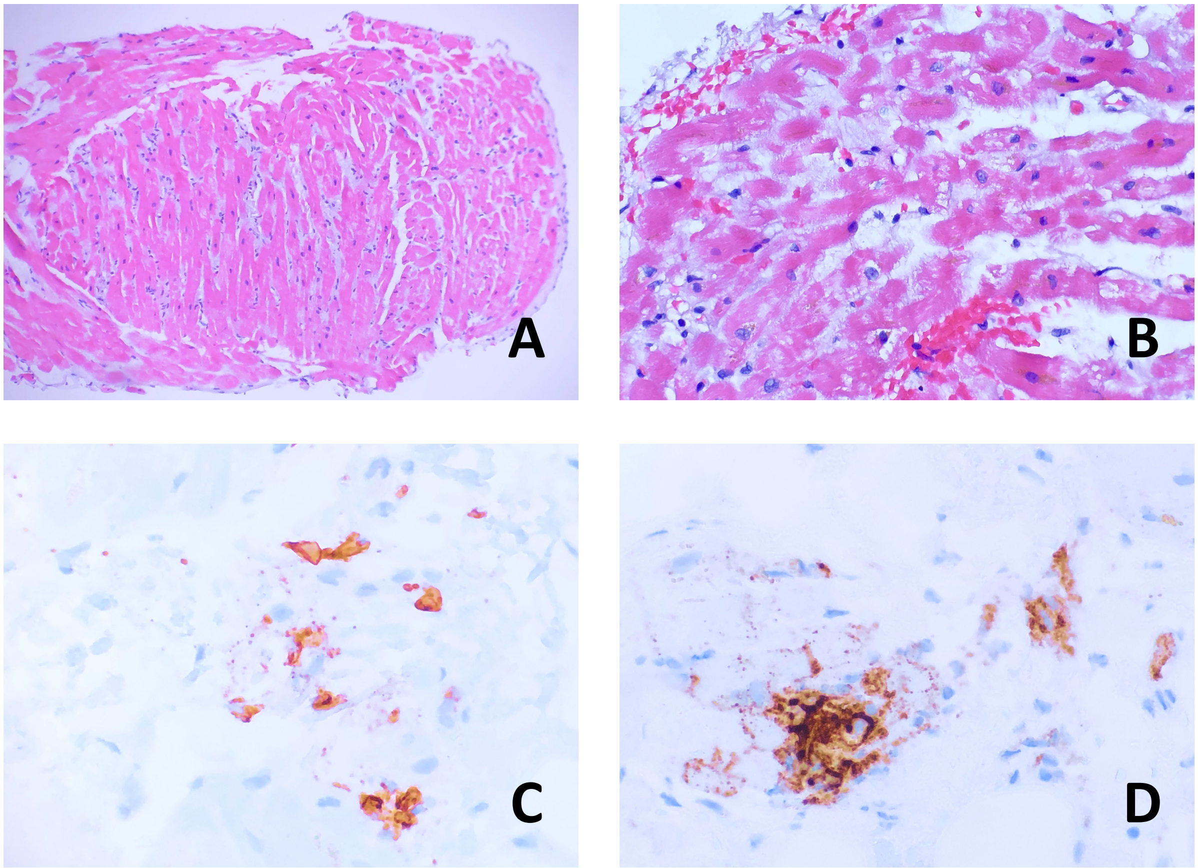

Supplemental Figure S1: Pathology Slides from Endomyocardial Biopsy Samples
A: Low power view of edematous endomyocardial biopsy fragments (H&E x100).
B: High power view of patchy myocyte damage (H&E x400).
C: Immunophenotyping highlights clusters of CD8+ T-cells in damaged tissue (x400).
D: Immunophenotyping is positive for PD-L1 (clone 22C3) within damaged foci (x400).
